# Frequency and risk factors of musculoskeletal pain in nurses at a tertiary centre in Jeddah, Saudi Arabia: a cross sectional study

**DOI:** 10.1186/1756-0500-7-61

**Published:** 2014-01-25

**Authors:** Suzan Mansour Attar

**Affiliations:** 1 Department of Internal Medicine, King Abdulaziz University, Jeddah, Kingdom of Saudi Arabia

**Keywords:** Pain, Nurse, Musculoskeletal

## Abstract

**Background:**

Musculoskeletal complaints are an important occupational problem; nevertheless, few studies have targeted nurses in Saudi Arabia. The aim of this study was to determine the frequency and risk factors of work-related musculoskeletal disorders (WMSDs) among nursing personnel at a tertiary centre in Jeddah.

**Methods:**

A comparative cross-sectional study was performed in which full-time registered nurses from four different departments (n = 200) were selected for analysis between September 1, 2011 and February 29, 2012. Musculoskeletal symptoms over the past year were assessed using the Nordic Standardised Musculoskeletal Questionnaire. In addition to demographic questions, the researcher evaluated employment history, physical risk factors at work, and general health status.

**Results:**

In this study, approximately 85% of the nurses reported experiencing at least one musculoskeletal symptom. Musculoskeletal symptoms occurred most commonly in the lower back (65.7%), ankles and feet (41.5%), and shoulders (29%). Prolonged working hours and being underweight were significantly associated with the development of these symptoms (OR 3.66, 95% CI 1.24-10.79, *P* = 0.018, and OR 2.66, 95% CI 1.37-5.93, *P* = 0.004, respectively). Working in the surgical department was a greater risk factor for low back pain compared with working in other departments.

**Conclusions:**

WMSDs are common among our nurses, and back pain is the most common symptom. As prolonged working hours and being underweight were factors that contributed most to WMSDs, decreasing shift durations or offering nutrition educational programmes may be suitable solutions. However, further studies are required to examine the best modality for decreasing the occurrence of WMSDs.

## Background

A work-related musculoskeletal disorder (WMSD) is defined as a musculoskeletal disorder that results from a work-related event [1]. WMSDs are common among health care personnel who provide patient care on a continuous basis. WMSDs are common among the nursing population worldwide, with a frequency of approximately 40 to 90% [2-6]. This statistic is not surprising given that the nursing population is exposed to continuous physical demands and musculoskeletal strains [7]. Because nurses represent approximately one-third of the working force at any hospital, the development of WMSDs in this population may have a substantial impact on absences from work, work restrictions, or even transfers to other jobs. We hypothesised that WMSDs might be more relevant in less-developed countries because of prolonged hospital stays and increased patient loads; however, to the best of our knowledge, no studies have explored the occurrence of different musculoskeletal complaints in nurses working at a hospital in Saudi Arabia [8,9]. Therefore, the aim of this study was to evaluate the frequency of WMSDs in the nursing population at a tertiary centre and to assess the relationship between WMSDs and workload-related factors.

## Methods

A comparative cross-sectional study was performed at a tertiary centre at King Abdulaziz University Hospital (KAUH) from September 1, 2011 to February 29, 2012, following approval by the Biomedical Ethical Research Committee of the Faculty of Medicine at King Abdulaziz University (KAU).

KAUH is a general teaching hospital in the western region of Saudi Arabia, with a total capacity of 800 beds. It is a major referral centre that provides tertiary care for patients in the Western Province of Saudi Arabia. The catchment area of the hospital includes approximately 350,000 people.

To be able to extrapolate our results, we calculated the sample size and performed a sampling technique to ensure a representative sample. The hospital has a database of 1074 nurses. At a confidence level of 95% with an alpha error = 0.05, the lowest reported frequency of WMSD is approximately 40% [2] and the maximum is 90% [3,5]; however, the frequency is assumed to range from 40% to 50% among nurses at KAUH. MedCalc statistical software was used to estimate a sample size of 200, which represents 20% of the working staff, as we could not examine all of the nurses. Using a simple random method, 200 nurses were selected from different hospital departments from a list of names obtained from the hospital administration and were interviewed by the researcher to complete a written questionnaire.

The nursing staff at KAUH consists of registered nurses with either a bachelor’s degree or diploma from their country of origin. Inpatient nurses work 48-hours weeks. Outpatient staff work according to the clinic’s needs, generally from 8 a.m. to 5 p.m. Employees other than nurses, including porters and unit clerks, were excluded from the study.

After obtaining formal informed consent from the participants, questionnaires were applied by the author. The questionnaires were divided into 3 different parts. The first part collected demographic data, including age, gender, marital status, and number of children. The second part consisted of perceptions of job risk factors that may contribute to the development of WMSDs, such as working in the Medicine, Surgery, Paediatric, or Obstetrics and Gynaecology departments. In addition, the questionnaire evaluated the hours at work, duration of employment in the current job in years, presence of co-morbid illness (diabetes mellitus, hypertension, chronic renal impairment, cardiovascular diseases, or malignancy), smoking, and participant weight (further divided according to the body mass index [BMI] into underweight [<18.5 kg/m^2^], healthy weight [18.5–24.9 kg/m^2^, overweight [25.0–29.9 kg/m^2^, and obese [≥30.0 kg/m^2^]).

The third portion of the questionnaire investigated WMSD. In this study we defined WMSD as musculoskeletal symptoms (pain, numbness, tingling, aching, stiffness, and burning) that resulted from a work-related event, excluding other injuries experienced over the past year that lasted 1 week or more or occurred at least monthly with at least moderate pain on average. It also assessed whether sick leave had been taken or medical advice had been sought. The researcher explained all the symptoms to the nurses and was available to answer any inquiry. The nurses responded yes or no to whether they had experienced these symptoms. This section was adapted from the previously validated and modified version of the Standardised Nordic Questionnaire of Musculoskeletal Symptoms (SNQ) that was established by Kuorinka *et al.* and is frequently used as to screen for MSDs [7,10]. The SNQ was used to evaluate nine body areas, including the three upper limb segments (shoulders, elbows, and wrists/hands), three lower limb segments (hips/thighs/buttocks, knees, and ankles/feet), and three trunk segments (neck, mid back, and lower back). We also evaluated the possible association of musculoskeletal complaints with different risk factors in the form of smoking habits and physical characteristics, in addition to the factors that increase the risk of WMSDs (such as the presence of insufficient nursing staff and manual lifting of patients).

### Statistical analysis

The data were analysed using the Statistical Package for the Social Sciences, version 16.0 software (SPSS Inc., Chicago, Illinois, USA). Quantitative data were presented in the form of means and standard deviations. Student’s t-test was used for comparisons between two groups, as the Kolmogorov-Smirnov test indicated that the data were parametric. Qualitative data were presented as frequencies and percentages. The chi-squared test (χ^2^) was used to compare qualitative data. Yates’ correction was used when the expected cell was less than five (< 5). The risk was estimated using odds ratio and 95% confidence interval. A multivariable linear regression analysis was performed to determine the predictors of WMSDs. A probability level of 0.05 or less was used to indicate statistical significance.

## Results

A total of 200 registered nurses were interviewed. Most of the nurses were females (191 females versus nine males). The mean age was 34.6 years (standard deviation [SD] ± 8.1 years). More than half (69%) of the nurses were married and worked 6 to 15 hours per day, with a mean of 12 hours (SD ± 1.1 hours) and a median of 12 hours. The total duration of employment ranged from 1 to 34 years (mean ± SD, 9 ± 6 years; median, 6 years). Table 1 shows the demographic features of the studied nurses.

**Table 1:** Descriptive information and risk factors affecting 200 studied nurses

**Variable**		**With WMSD n=169(%)**	**Without WMSD n=31(%)**	**OR**	**95% CI**	***P*-value**
**Age (yrs): Mean±SD (range)**		34.9±8.1 (22–57)	33.42±8 (22–56)			NS^$^
**Gender**						
**Male**	**n = 9**	8/161	1/30	1.4	(0.18–32.99)	NS
**Female**	**n = 191**			0.67	(0.03–5.64)	
**Marital status**						
**Married**	**n = 139**	118 (85)	21 (15)	1.1	(0.5–2.5)	NS
**Not married**	**n = 61**	51 (84)	10 (16)			
**Children**						
**Yes**	**n = 104**	87 (84)	17 (16)	0.95	(0.42–2.15)	NS
**No**	**n = 96**	81 (85)	15 (15)		Reference category	
**Department**						
**Medicine**	**n = 73**	61 (64)	12 (16)	0.89	(0.38–2.11)	NS
**Surgery**	**n = 45**	41 (91)	4 (9)	**2.16**	(0.67–7.77)	NS
**Paediatric**	**n = 42**	36 (86)	6 (14)	1.13	(0.4–3.33)	NS
**Obstetrics &Gyn**a**ecology**	**n = 40**	31 (77)	9 (23)	0.55	(0.21–1.43)	NS
**Working hours per day**						
**5–10 hours**	**n = 20**	13 (65)	7 (35)		Reference category	
**More than 10 hours**	**n = 180**	156 (87)	24 (13)	**3.5**	(1.13–10.67)	0.01**
**Duration of job (yr)**						
**Less than 1**	**n = 21**	15 (71)	6 (29)		Reference category	
**Between 1–5**	**n = 72**	60 (83)	12 (17)	**2**	(0.56–7.06)	NS
**Between 6–10**	**n = 38**	34 (90)	4 (10)	**3.4**	(0.7–17.3)	NS
**More than 10**	**n = 69**	60 (87)	9 (13)	**2.67**	(0.71–10.01)	NS
**Co–morbid illness**						
**Yes**	**n = 49**	43 (87)	6 (13)	1.42	(0.51–4.16)	NS
**No**	**n = 151**	126 (83)	25 (17)		Reference category	
**Weight**						
**Underweight**	**n = 67**	50 (75)	17 (25)	0.45	(0.19–1.07)	0.05*
**Normal weight**	**n = 100**	87 (87)	13 (13)		Reference category	-
**Overweight**	**n = 24**	24 (100)	-	0.82	(0.77–0.88)	NS
**Obese**	**n = 9**	8 (89)	1 (11)	1.5	(0.18–12.4)	NS
**Smoking**						
**Yes**	**n = 3**	2 (75)	1 (25)	1.9	(0.64–2)	NS
**No**	**n = 197**	165 (84)	32 (16)		Reference category	
**Work-related factors**						
** Manual lifting of patients**						
**Yes**	**n = 177**	152 (86)	25 (14)	**2.15**	(0.68–6.5)	NS
**No**	**n = 23**	17 (73)	6 (27)		Reference category	
** Not enough staff**						
**Yes**	**n = 112**	101 (90)	11 (10)	**2.7**	(1.14–6.47)	0.01**
**No**	**n = 88**	68 (77)	20 (23)		Reference category	

**WMSDs**: Work-related musculoskeletal disorders**, yr:** years**, OR:** Odds ratio.

***P*-value** was obtained using the chi-squared test and student’s t-test^$^.

Significant at <0.05*, *P* < 0.01** and *P* < 0.001***.

Table 2 shows the frequency of WMSDs in the study sample as a whole. The overall 12-month prevalence of self-reported WMSDs was 85% (95% CI 79-89%). Lower back pain (LBP) was the most commonly reported WMSD, with a frequency of 65.7% (95% CI 58.7-72.1%). Symptoms of the wrist (10%, 95% CI 6.4-15.2%), mid-back (5%, 95% CI 2.5-9.7%), and elbow (3%, 95% CI 1.2-6.7%) were the least common.

**Table 2:** Frequency of work-related musculoskeletal disorders among 200 studied nurses

**WMSDs**	**Frequency**	**Percentage**
**Total symptoms**	**169**	**85**
**Trunk**		
**Neck**	**40**	**20**
**Mid-back**	**10**	**5**
**Lower back**	**130**	**65.7**
**Upper limb**		
**Shoulder**	**58**	**29**
**Elbow**	**6**	**3**
**Wrist/hand**	**20**	**10**
**Lower limb**		
**Hip/thigh/buttocks**	**33**	**16.5**
**Knee**	**42**	**21**
**Ankle/foot**	**83**	**41.5**

**WMSDs**: Work-related musculoskeletal disorders**, CI:** confidence interval.

The results of the logistic regression analyses that explored the most significant risk factors for WMSDs are shown in Table 3. Multivariable linear regression analysis indicated that nurses who worked more than 10 hours per day had an increased risk of WMSDs (OR 3.66, 95% CI 1.24-10.79, *P* = 0.018) compared with nurses who did not. Being underweight was also a significant risk factor (OR 2.66, 95% CI 1.37-5.93, *P* = 0.004).

**Table 3:** Multivariable linear regression analysis for predicting the most significant risk factor for work-related musculoskeletal disorders (WMSDs)

**Variable**	***P*-value**	**Adjusted OR**	**(95% CI)**
**Weight**			
**-Underweight**	0.01**	2.66	(1.37–5.93)
**Working hours per day**			
**-More than 10 hours**	0.01**	3.66	(1.24–10.79)

**WMSDs**: Work-related musculoskeletal disorders**, OR:** Odds ratio, **CI:** Confidence interval.

*P*- value obtained using logistic regression analysis.

Significant at <0.05*, *P* < 0.01** and *P* < 0.001***.

Because LBP was the most common symptom in 130 patients (65%), the author explored the risk factors that contributed to this effect. Table 4 includes the risk factors associated with LBP among nurses. The greatest risk was among nurses working in the Surgical Department (OR 2.2, 95% CI 1–4.8, *P* = 0.041). The lowest frequency was recorded among nurses who worked in the Obstetrics and Gynaecology Department (OR 1.5, 95% CI 1–2.1, *P* = 0.009).

**Table 4:** Risk factors associated with the presence of lower back pain among nurses

**Variable**	**With LBP n = 130(%)**	**Without LBP n = 70(%)**	***X*** **2**	**Unadjusted OR (95% CI)**	***P*-value**
**Department**					
**Medicine n = 73**	52 (40)	21 (30)	2	1.56 (0.84–2.9)	NS
**Surgery n = 45**	35 (27)	10 (14)	4.2	2.2 (1–4.8)	<0.05*
**Paediatric n = 42**	24 (18)	18 (26)	2.2	1.2 (0.9–1.66)	NS
**Ob & Gyn n = 40**	19 (15)	21 (30)	6.73	1.5 (1–2.1)	<0.01**
**Manual lifting of patients**					
**Yes n = 177**	116 (65)	61 (35)	0.2	1.2 (0.5–2.9)	NS
**No n = 23**	14 (61)	9 (39)			
**Not enough staff**					
**Yes n = 112**	75 (67)	37 (33)	0.43	1.2 (0.7–2.2)	NS
**No n = 88**	55 (62)	33 (38)			

**LBP:** Low back pain, **OR:** Odds ratio, NS : non significant.

***P*-value** were obtained using the chi-squared test.

*Significant at <0.05*, *P* < 0.01** and *P* < 0.001***.

## Discussion

In this cross-sectional survey of 200 nurses, WMSDs were observed in 169 patients (85%). The most common site affected was the lower back in 130 patients (65.7%), followed by the ankle/foot in 83 patients (41.5%) and shoulder in 58 patients (29.0%). The most significant risk factors were prolonged shifts (>10 hours per day) and being underweight.

Previous studies have documented various rates of WMSDs over a 12-month period in a variety of populations. Data from the present study were comparable to the that reported from Nigeria (85%) [1], but higher than those observed in Mexico (76%) [11], Japan (70%) [6], Canada (66%) [12], and the United States (60%) [2]. On the contrary, the rate was lower than that in Brazil (93%) [3] and Turkey (90%) [5]. The results of these studies should be interpreted carefully, and the standard questions should be evaluated to determine whether diverse symptoms (pain, numbness, tingling, aching, stiffness, and burning) rather than one symptom alone (pain) were assessed, as this contributes to a higher frequency. The present study included all possible associated symptoms and used the Standardised Nordic Questionnaire of Musculoskeletal Symptoms (SNQ), which has been included in other studies [1-4,11,13].

This distribution pattern of WMSDs among the nursing population is consistent with that reported in the literature, as LBP was the most common complaint (65.7%) in our nursing population. Previous studies have documented different rates of LBP over a 12-month period, ranging between 19 and 80%. The highest rates, which were consistent with our finding, were from Greece (75%) [4], Nigeria (73.5%) [14], Germany (73%) [15], Turkey (69%) [5], Sweden (64%) [16], and Italy (60%) [17]. In other studies, LBP was still the most common WMSD, but the frequency was not comparable to our findings. In these reports, the rates of LBP were 59% in Australia [18], 56% in China [13], 48% in Canada [19], 40% in England [20], 41.1% in France [21], 40.6% in Hong Kong [22], and 29% in the United States [23].

Back pain is the most common WMSD among all populations because of its significant association with locally stressful physical activities. This study attempted to explore this association, which was significantly associated with the Surgical Department. This may be related to the need for increased manual handling of postoperative patients within a short period of time. Lorusso *et al.* ‘;s recent systematic review of 25 studies that examined the prevalence of LBP among Italian nurses concluded that psychosocial risk factors associated with physical workload were significantly associated with LBP in most studies [17]. Similarly, Tezel *et al.* observed that WMSDs were significantly associated with working in Surgical and Obstetrics and Gynaecology departments, where nurses were more likely to have chronic WMSDs than nurses working in other departments [5]. However, in our study, the frequency of WMSDs was lowest (47%) amongst nurses who worked in the Obstetrics and Gynaecology Department compared to those who worked in the other departments.

The period-prevalence of ankle and foot WMSDs among nurses was 41.5% (95% CI 34.6-48.7%). Compared to other developing countries, this value was higher, with studies reporting a frequency of 10.2 to 16.6% [1,11]. Numerous conditions can cause chronic foot and ankle pain, but pain is primarily caused by inappropriate footwear [24]. Flexible flat feet is a pathological foot condition that can occur in older nurses [25]. Because nurses spend most of their working time on their feet, they may develop numerous conditions that result from wearing inappropriate footwear that can cause chronic foot and ankle pain, such as plantar fasciitis, bunions, and hammer toes. Further questioning of the nurses revealed that 64/83 (77%) were unaware of the causes of their foot pain, as they never sought medical attention.

Shoulder pain was the third most commonly reported WMSD within the studied cohort, with a period-prevalence of 29%, which was lower than the values reported from Sweden (60%) [26], Australia (60%) [16], South Africa (41%) [27], and the United States (35.1%) [2].

Two risk factors contributed to the development of WMSDs in our nurses, namely working more than 10 hours per day and being underweight. Working more than 10 hours per day causes both physical and psychological stress. Work stress and WMSDs have been a recent area of interest for researchers. A systematic review conducted in the Netherlands showed that job-related stress was a significant risk factor for upper extremity pain in both high- and low-quality studies [28]. In addition, back and leg complaints have been positively associated with hours worked per week, especially among nurses working more than 35 hours/week [29]. A decrease in the recovery time between shifts results in an increase in the expression of pain [23]. Workloads and stress have been associated not only with pain but also with injuries. Clark *et al.* found that high workloads were associated with a 50 to 200% increase in the likelihood of needle stick injuries and near misses among hospital nurses [30].

Prior studies have demonstrated a strong association between being overweight and WMSDs; however, this report showed that being underweight has a strong and significant association with WMSDs [31]. It is possible that this finding indicates that being underweight is related to a lack of physical strength [32].

Our nursing population is similar to the other nursing populations in other health facilities in the Kingdom. As the Kingdom relies on the same nursing workforce consisting of nurse of similar age groups, nationalities and racial backgrounds, the only difference lies in their geographical distribution. As a result, it is possible to generalize our results to other nursing populations across the Kingdom.

The advantage of our study is that it explores all pain-related complaints, while other studies explored only pain [1,14,17]. In addition, our study includes multiple body parts, unlike other studies that included only one site [14,15,28]. One disadvantage of the present study is the possibility of incomplete symptom recall, particularly if the last symptom occurred many months before the questionnaire was completed. Nevertheless, the study duration was based on previous similar studies.

## Conclusion

The present study indicated that WMSDs are common among nurses at a tertiary care centre in Saudi Arabia, and the frequency of symptoms is consistent with that reported from other facilities. Risk factors that contributed to the development of WMSDs were prolonged shifts and being underweight, which are factors that may be difficult to avoid. As a result, it may be beneficial for hospital administrations to adopt certain strategies, such as shorter shifts in the most at-risk departments, programs emphasising proper nutrition, or the inclusion of additional breaks to allow nurses to eat. Future studies are needed to evaluate the most effective methods for WMSD prevention. For now, special referrals are performed for certain cases with persistent musculoskeletal symptoms. Because working in the obstetrics and gynaecology department is considered protective for back pain, a follow-up will be performed to determine which strategies are used by this department and how these strategies can be shared with the rest of the hospital departments.

## Competing interest

The author declare that she have no competing interests.
